# CO_2_ Capture in the Sustainable Wheat-Derived Activated Microporous Carbon Compartments

**DOI:** 10.1038/srep34590

**Published:** 2016-10-04

**Authors:** Seok-Min Hong, Eunji Jang, Arthur D. Dysart, Vilas G. Pol, Ki Bong Lee

**Affiliations:** 1 Department of Chemical and Biological Engineering, Korea University, 145 Anam-ro, Seongbuk-gu, Seoul 136-713, Republic of Korea; 2 School of Chemical Engineering, Purdue University, 480 Stadium Mall Drive, West Lafayette, Indiana 47907-2100, United States

## Abstract

Microporous carbon compartments (MCCs) were developed via controlled carbonization of wheat flour producing large cavities that allow CO_2_ gas molecules to access micropores and adsorb effectively. KOH activation of MCCs was conducted at 700 °C with varying mass ratios of KOH/C ranging from 1 to 5, and the effects of activation conditions on the prepared carbon materials in terms of the characteristics and behavior of CO_2_ adsorption were investigated. Textural properties, such as specific surface area and total pore volume, linearly increased with the KOH/C ratio, attributed to the development of pores and enlargement of pores within carbon. The highest CO_2_ adsorption capacities of 5.70 mol kg^−1^ at 0 °C and 3.48 mol kg^−1^ at 25 °C were obtained for MCC activated with a KOH/C ratio of 3 (MCC-K3). In addition, CO_2_ adsorption uptake was significantly dependent on the volume of narrow micropores with a pore size of less than 0.8 nm rather than the volume of larger pores or surface area. MCC-K3 also exhibited excellent cyclic stability, facile regeneration, and rapid adsorption kinetics. As compared to the pseudo-first-order model, the pseudo-second-order kinetic model described the experimental adsorption data methodically.

With increasing CO_2_ emissions, global warming is accelerated, accompanied by abnormal climate changes^1^,^2^. According to the 2015 Paris Climate Conference, the global agreement for the reduction of CO_2_ emissions was strengthened^3^. Although most of the emitted CO_2_ has been produced from the combustion of fossil fuels, it is inevitable to use fossil fuels as major energy sources because of their availabilities and economic efficiencies, resulting in high demand^4^. For decreasing CO_2_ emissions, carbon dioxide capture and sequestration (CCS) is considered as one of the promising technologies. In the CCS technology, CO_2_ capture accounts for the largest portion of the total cost; thus, it is imperative to develop efficient methods for capturing CO_2_^5^. Various approaches have been applied for capturing CO_2_, such as absorption, adsorption, and membrane separation. Among these approaches, absorption by using liquid amines has been widely used for capturing CO_2_ because of its large capacity and facile application. However, absorption requires enormous energy during regeneration as well as produces polluted products^6^. On the other hand, membrane separation is a simple, continuous operation; however, it suffers from a drawback of limited performance in case of low CO_2_ concentrations^7^. Recently, adsorption is considered to be an efficient technology for capturing CO_2_ because of its low energy consumption and facile regeneration, without the production of pollution or by-products^8^.

Zeolites and metal–organic frameworks (MOFs) are widely used commercial CO_2_ adsorbents, but they suffer from a drawback of degradation of the CO_2_ adsorption ability under humid conditions^9^. However, porous carbons can be used for the adsorption of CO_2_ even under humid conditions, attributed to their hydrophobic properties; they also exhibit advantages of large surface area with high porosity, rapid adsorption–desorption kinetics, thermal and chemical stability, as well as cost-effective preparation^10^.

Moreover, the characteristics of porous carbon adsorbents can be varied by the use of carbon precursors and activation methods. Activation by physical or chemical treatment is conducted for developing porosities in carbonaceous materials; in physical activation, carbon is partially gasified using oxidising gases, such as steam, CO_2_, and air^11^,^12^,^13^. On the other hand, chemical activation is conducted by chemical reaction between carbonaceous materials and chemical agents, such as KOH, NaOH, K_2_CO_3_, ZnCl_2_, and H_3_PO_4_^14^,^15^,^16^,^17^,^18^. Generally, as compared to physical activation, chemical activation is favored because it creates a well-developed microporous structure of carbon^19^. Also, several studies have attempted to correlate CO_2_ adsorption capacity and textural properties, such as surface area and pore volume, for activated porous carbon^20^,^21^,^22^. Recent studies have suggested that CO_2_ adsorption capacity at ambient temperature and pressure is closely related to the narrow micropore volume of the adsorbent^23^,^24^.

Carbonaceous materials are derived from various carbon precursors, such as polymer, biomass, coal, petroleum residues, and bones^25^,^26^,^27^,^28^,^29^. Unur *et al.* have prepared nanoporous carbon derived from hazelnut shells via carbonization and KOH activation^30^. The obtained porous carbon exhibited a high specific surface area of 1700 m^2^ g^−1^ and pore volume of 0.79 cm^3^ g^−1^, the values of which are approximately five times greater than those of carbon without activation. Kante *et al.* have synthesized coffee-based activated carbon by ZnCl_2_ activation, with a specific surface area and total pore volume of 1121 m^2^ g^−1^ and 0.95 cm^3^ g^−1^, respectively^31^. The textural properties of the samples thus prepared significantly depend on the ZnCl_2_/C ratio.

In this study, for preparing novel CO_2_ adsorbents, activated microporous carbon was prepared from the carbonization of wheat flour, followed by KOH activation. Wheat contains various polysaccharides, such as starch, cellulose, glucose, and xylose, which are released from carbonaceous structures when wheat is pyrolyzed at high temperature^32^,^33^,^34^,^35^. To the best of our knowledge, no studies have been reported about the application of wheat-based porous carbon for CO_2_ capture. In our study, varying KOH/C ratios were applied during activation, and the effects of the KOH/C ratio on the textural properties and CO_2_ adsorption abilities of the prepared porous carbon were investigated. In addition, the relationship between the textural properties of wheat-based porous carbon and its CO_2_ adsorption capacity was investigated, and various characteristics of CO_2_ adsorption, such as isosteric heat of adsorption, selectivity of CO_2_ over N_2_, adsorption kinetics, and cyclic stability, were investigated.

## Results and Discussion

### Characteristics of KOH-activated MCCs

**Figure 1:**
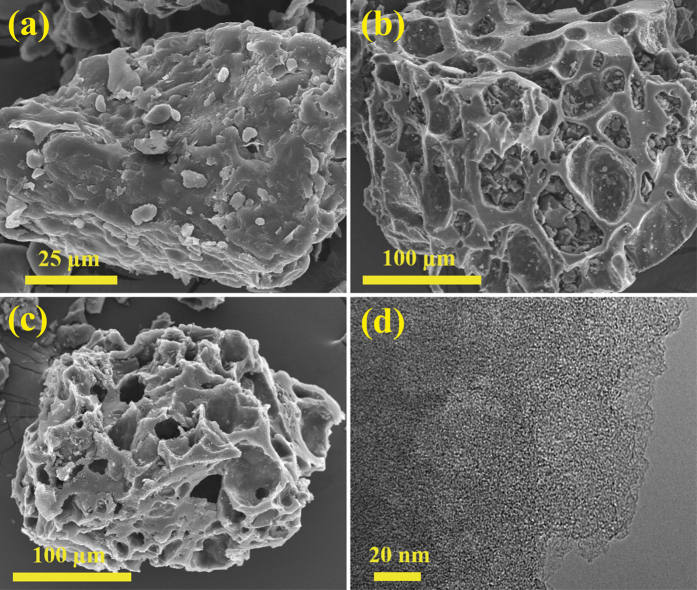
SEM images of (**a**) pristine wheat flour, (**b**) MCC, and (**c**) MCC-K3. (**d**) TEM image of MCC-K3.

Figure 1 shows the morphologies of pristine wheat flour, microporous carbon compartments (MCCs) and MCC activated with a KOH/C ratio of 3 (hereafter, referred to as MCC-K3). As shown in Fig. 1a, pristine wheat flour exhibited large irregular-shaped chunks. After carbonization, wheat flour changed to MCC, which is composed of flakes forming big compartments; these compartments contribute to high surface area and pore volume (Fig. 1b). After activation, MCC did not exhibit any significant change in morphology (Fig. 1c). As shown in the TEM image (Fig. 1d), MCC-K3 exhibited worm-like micropores distributed randomly through the carbon. Figure S1 (Supplementary Information) shows the XRD spectra of the porous carbon materials thus obtained, which are regarded as amorphous carbon.

Figure S2 (Supplementary Information) shows the thermogravimetric analysis (TGA) of pristine wheat flour. At or less than 100 °C, pristine wheat flour exhibited a weight loss of approximately 7.6 wt%, attributed to water vapour or steam. In addition, weight started to decrease at ~200 °C, and a substantial weight decrease was observed at around 317 °C. During carbonization, a significant amount of non-carbon elements are believed to be pyrolyzed, with the simultaneous development of pores. Figure S3 (Supplementary Information) shows the Fourier transform infrared (FTIR) spectra of pristine wheat flour, MCC, and MCC-K3, confirming the decomposition of these foreign components. Pristine wheat flour exhibited peaks related to various functional groups. A broad band was observed at 3293 cm^−1^, attributed to hydroxyl O–H, while bands were observed at 2926 and 2857 cm^−1^, attributed to C–H stretching, and at 1742 cm^−1^, attributed to C = O stretching vibration^36^. Peaks were also observed at 1648 and 1536 cm^−1^, characteristic of stretching vibrations of C–O from amide I and C–N from amide II, respectively^37^, while peaks observed at 1149 and 1014 cm^−1^, attributed to C–O stretching vibration^36^,^38^. However, no noticeable peaks were observed in the spectra of MCC and MCC-K3, suggesting that the characteristic functional groups of non-carbon elements disappear during carbonization.

**Figure 2:**
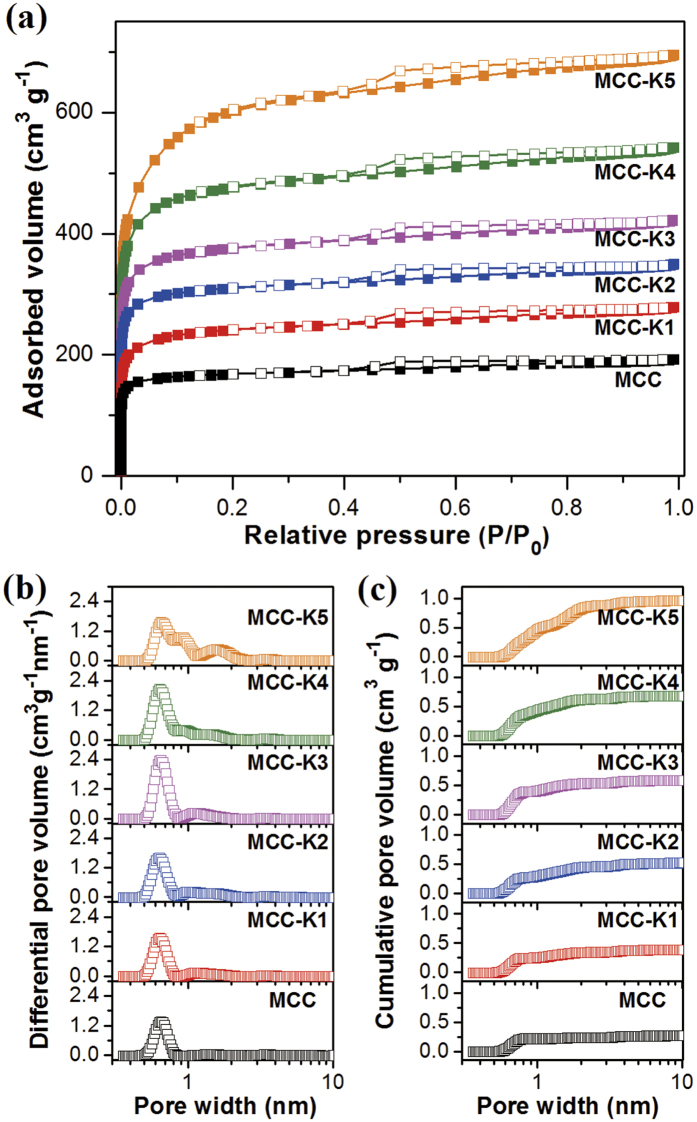
(**a**) N_2_ adsorption isotherms at 77 K, (**b**) pore size distribution, and (**c**) cumulative pore volume of MCC and KOH-activated MCCs.

Figure 2a shows the N_2_ adsorption isotherms at 77 K for MCC and KOH-activated MCCs. According to the IUPAC classification, the samples exhibited type I isotherms, indicative of microporous structures^39^. With increasing KOH/C ratio, the amount of adsorbed N_2_ increased because of the development of pores. Pore size distribution was estimated using N_2_ adsorption data and non-local density functional theory (NLDFT), as shown in Fig. 2b. MCC mainly exhibited narrow micropores with a size of 0.5–0.8 nm; this size is known to be favorable for the adsorption of CO_2_^40^. More narrow micropores with a pore size of less than 0.8 nm were newly developed as more KOH was consumed during activation up to a KOH/C ratio of 3. However, additional larger pores with a size of 0.8–2.0 nm were simultaneously observed for KOH-activated MCCs, attributed to the further reaction of small-sized pores in carbon with KOH, resulting in enlarged pores. As can be clearly observed for highly activated MCC (KOH/C = 4 or 5), when a large amount of KOH was used for activation, the number of enlarged pores became significantly greater than that of newly developed small-sized pores. Hence, the narrow micropore volumes of MCC-K4 and MCC-K5 are less than that of MCC-K3.

Table 1 summarizes the textural properties obtained from N_2_ adsorption isotherms at 77 K. MCC exhibited a high specific surface area of 648 m^2^ g^−1^ and a total pore volume of 0.299 cm^3^ g^−1^, indicating potential for applications as an adsorbent. The textural properties of MCC were further enhanced by KOH activation. With increasing KOH/C ratio during KOH activation, the textural properties gradually increased, and the highest specific surface area of 2192 m^2^ g^−1^ and total pore volume of 1.076 cm^3^ g^−1^ were obtained for MCC-K5. Also, the volume of narrow micropores having a size of less than 0.8 nm was estimated from the cumulative pore volume (Fig. 2c). The volume of the narrow micropores increased with increasing KOH/C ratio until 3, followed by the decrease in volume for MCC-K4 and MCC-K5, attributed to pore enlargement.

**Figure 3:**
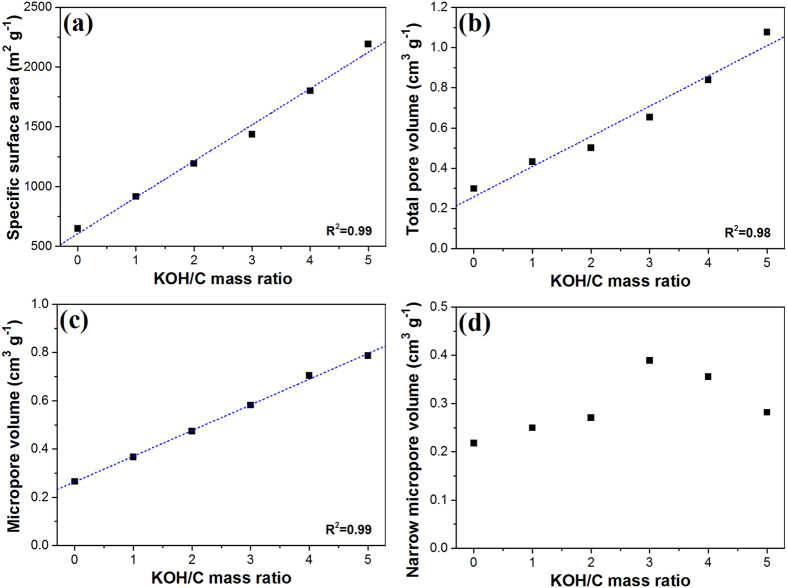
Relationship between the KOH/C ratio and textural properties, such as (**a**) specific surface area, (**b**) total pore volume, (**c**) micropore volume, and (**d**) narrow micropore volume.

Figure 3 shows the relationship between the KOH/C ratio during activation and the textural properties of samples. With increasing KOH/C ratio, new micropores were developed, and the size of pores enlarged, resulting in increased specific surface area, total pore volume, and micropore volume. According to the calculated linearity (coefficient of determination, R^2^ > 0.98), the specific surface area, total pore volume, and micropore volume of MCC were highly correlated with the amount of KOH used during activation. However, poor correlation was observed between the KOH/C ratio and volume of narrow micropores, with a pore size of less than 0.8 nm, because excess of KOH (KOH/C = 4 or 5) widened pores, eventually decreasing the narrow micropore volume.

### CO_2_ adsorption on KOH-activated MCCs

Table 1 also summarizes the CO_2_ adsorption capacities at 0, 25, 50, and 75 °C. MCC exhibited CO_2_ adsorption capacities of 3.44 mol kg^−1^ and 2.28 mol kg^−1^ at 0 °C and 25 °C, respectively, and after KOH activation, the adsorption capacities gradually increased with increasing KOH/C ratio up to 3, with the highest CO_2_ adsorption capacity of 5.70 mol kg^−1^ and 3.48 mol kg^−1^ at 0 °C and 25 °C, respectively, for MCC-K3. The CO_2_ adsorption capacity of MCC-K3 was higher or comparable to those reported recently for porous carbons, such as N-doped carbon and porous carbon activated by KOH, CO_2_, or steam (Table S1, Supplementary Information)^41^,^42^,^43^,^44^,^45^,^46^,^47^,^48^,^49^,^50^,^51^,^52^. When excess of KOH was used for activation, the CO_2_ adsorption capacity rather decreased to 3.13 and 2.56 mol kg^−1^ at 25 °C for MCC-K4 and MCC-K5, respectively.

**Figure 4:**
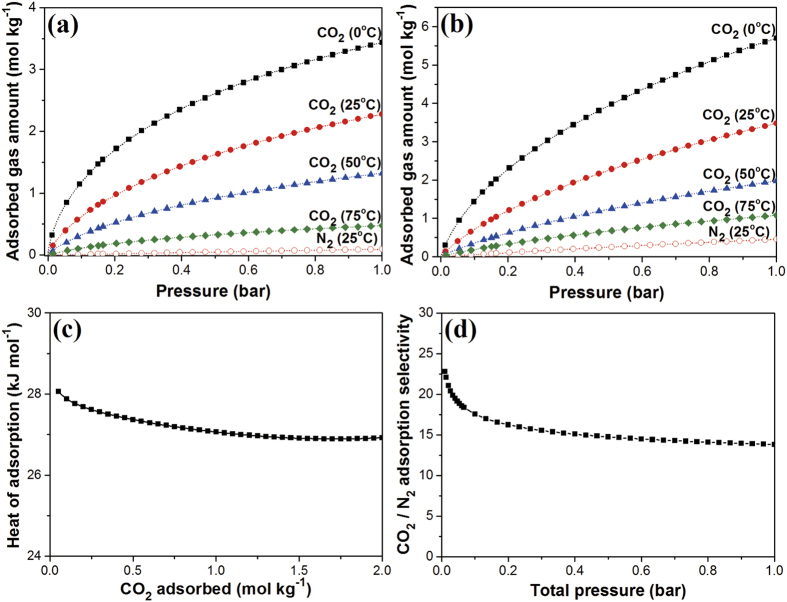
CO_2_ adsorption isotherms at 0, 25, 50, and 75 °C, and N_2_ adsorption isotherm at 25 °C of (**a**) MCC and (**b**) MCC-K3. Symbols and dashed lines represent experimental data and fitted results, respectively. (**c**) Isosteric heat of adsorption and (**d**) IAST-predicted adsorption selectivity of CO_2_ over N_2_ at 25 °C for a CO_2_/N_2_ binary gas mixture (CO_2_/N_2_ = 15:85) for MCC-K3.

Figure 4a,b show the adsorption isotherms of CO_2_ and N_2_ for MCC and MCC-K3, respectively. Adsorption isotherms fitted well with the dual-site Langmuir–Freundlich model. High CO_2_ adsorption capacities were obtained at low temperature, implying that the adsorption of CO_2_ on MCC and MCC-K3 corresponds to exothermic physisorption. As shown in Figure S4 (Supplementary Information), other KOH-activated MCC samples also exhibited similar trends. In addition, notably, MCC and MCC-K3 exhibited higher adsorption capacities for CO_2_ as compared to N_2_, attributed to the high quadrupole moment and polarizability of CO_2_ molecules, which in turn induce stronger interaction between the carbon structure and CO_2_ as compared to N_2_^53^.

Figure 4c shows the isosteric heat of adsorption calculated from the Clausius–Clapeyron equation for MCC-K3 using CO_2_ isotherms at 0, 25, and 50 °C^54^. Under low adsorption coverage, isosteric heat was as high as 28.1 kJ mol^−1^, attributed to the strong interaction between CO_2_ molecules and the most favorable active sites on porous carbon. However, with increasing adsorption coverage, the favorable active sites were consecutively occupied by CO_2_, and the isosteric heat of adsorption decreased to 26.9 kJ mol^−1^, accompanied with weak interactions between adsorbent and adsorbate. The calculated isosteric heat of adsorption varied in the range of ordinary physisorption (<40 kJ mol^−1^); hence, facile desorption is expected during regeneration.

Typically, the gas emitted from the combustion of fossil fuels contains approximately 15% CO_2_ with mostly balanced N_2_. Hence, it is imperative that the adsorbent exhibits high selectivity for CO_2_ over N_2_. Selectivity can be calculated by the ideal adsorption solution theory (IAST) using isotherms of CO_2_ and N_2_ at 25 °C^55^. The IAST-predicted selectivity of MCC-K3 for a 15% CO_2_ and 85% N_2_ mixture was 15–23 (Fig. 4d). CO_2_/N_2_ selectivity determined by applying an initial slope calculation method was ~16 (Figure S5, Supplementary Information); these selectivity values are comparable to those of porous carbon reported recently (Table S1, Supplementary Information). The selectivity of CO_2_ over N_2_ can be further enhanced by amine modification or heteroatom doping^56^,^57^,^58^,^59^.

**Figure 5:**
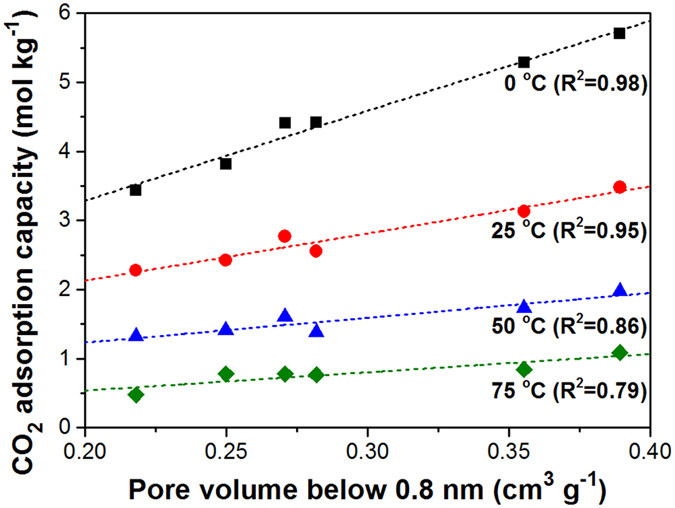
Correlation between CO_2_ adsorption capacities and volume of narrow micropores with a pore size of less than 0.8 nm.

For better understanding the effect of textural properties on CO_2_ adsorption, the CO_2_ adsorption capacities measured at 0, 25, 50, and 75 °C were correlated with the specific surface area, total pore volume, micropore volume, and volume of narrow micropores with a pore size of less than 0.8 nm for KOH-activated MCCs. The specific surface area, total pore volume, and micropore volume were not related to CO_2_ adsorption capacities (Figure S6, Supplementary Information). However, the volume of the narrow micropores with a pore size of less than 0.8 nm exhibited very close correlation with CO_2_ adsorption capacities, having high R^2^ values (Fig. 5). This result is in agreement with those reported in recent studies, suggesting that the narrow micropore volume plays a significant role in determining CO_2_ adsorption performance^60^,^61^,^62^. The CO_2_ adsorption capacity has been reported to be primarily affected by the number of narrow micropores because the van der Waals force from the surrounding walls of narrow micropores is thought to provide favorable interaction between the carbon structure and CO_2_ molecules^63^. Hence, for the efficient adsorption of CO_2_, it is imperative to develop narrow micropores of less than 0.8 nm in porous carbon by controlling the KOH/C ratio.

As important characteristics for CO_2_ adsorbents, adsorption kinetics, cyclic stability, and ease of regeneration are as important as high CO_2_ adsorption capacity. For evaluating adsorption kinetics, the adsorption of CO_2_ on MCC-K3 was measured by TGA at 30, 40, and 50 °C under atmospheric pressure. After the start of CO_2_ gas flow, CO_2_ adsorption uptake reached 50% of the equilibrium capacity within 2 min and 70% within 5 min, indicating a rapid CO_2_ adsorption rate. In addition, for describing the CO_2_ adsorption rate of MCC-K3, the widely used pseudo-first-order and pseudo-second-order models were applied. The pseudo-first-order model is based on the adsorption rate, which is proportional to the number of possible adsorption sites, and it can be expressed as follows:


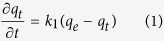


Here, *q*_*e*_ and *q*_*t*_ denote the adsorption uptake at equilibrium and at certain time *t*, respectively, and *k*_1_ is the pseudo-first-order adsorption rate constant. By applying the boundary conditions of *q*_*t*_ = 0 at *t* = 0 and *q*_*t*_ = *q*_*e*_ at *t* = ∞, the integrated form of equation can be written as follows:


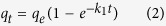


The pseudo-second-order model is based on the assumption that the adsorption rate is proportional to the square of the number of possible adsorption sites, and the adsorption rate can be expressed as follows:


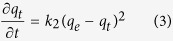


Here, *k*_2_ is the pseudo-second-order adsorption rate constant. By integrating the rate equation with the same boundary conditions of *q*_*t*_ = 0 at *t* = 0 and *q*_*t*_ = *q*_*e*_ at *t* = ∞, equation (3) can be rearranged as follows:


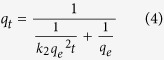


**Figure 6:**
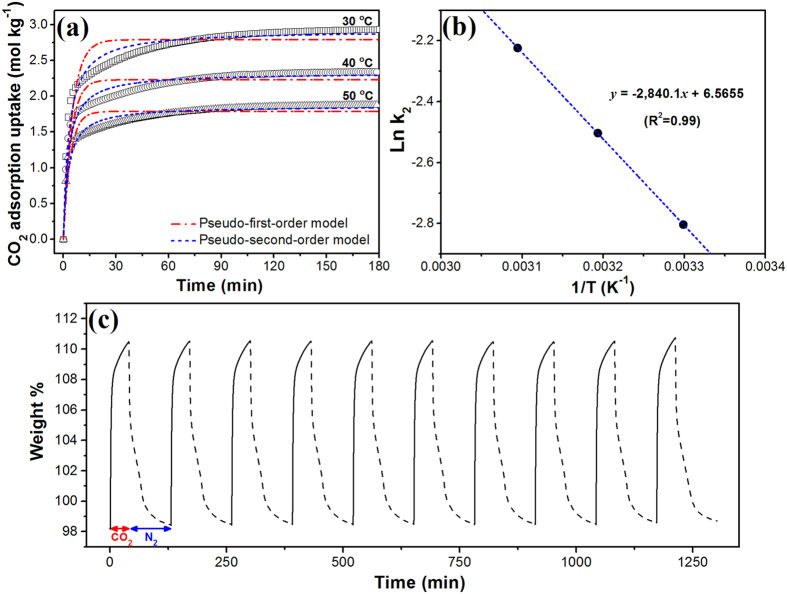
(**a**) CO_2_ adsorption kinetics for MCC-K3. Symbols and dashed lines denote experimental data and model fittings, respectively. (**b**) Arrhenius plot of pseudo-second-order adsorption rate constants for the estimation of activation energy of CO_2_ adsorption on MCC-K3. (**c**) Cyclic stability test for MCC-K3. Solid and dashed lines represent the adsorption and desorption steps, respectively.

Figure 6a shows the fitted curves using the pseudo-first-order and pseudo-second-order kinetic models, and Table 2 summarizes the related parameters. As compared to the pseudo-first-order model (R^2^ = 0.70–0.72), the pseudo-second-order model well fitted the experimental data (R^2^ = 0.92–0.96) at various adsorption temperatures. The small normalized standard deviation (Δ*Err*%) also confirms that the pseudo-second-order model is more suitable than the pseudo-first-order model for describing experimental data.

Basically, rate constants are dependent on temperature, and temperature dependency can be described by the Arrhenius equation as follows:


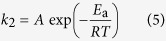


Here, *A* is the Arrhenius pre-exponential factor, *E*_a_ is the apparent activation energy, *R* is the universal gas constant and *T* is the absolute temperature. Figure 6b shows the linear plot of the natural logarithm of *k*_2_ against reciprocal temperature, which exhibited reasonable linearity. From the slope and intercept of the linear plot, MCC-K3 exhibited an *E*_a_ of 23.61 kJ mol^−1^ and *A* of 710.17 mol kg^−1^ min^−1^. Activation energy ranged from 5 to 40 kJ mol^−1^, indicating that CO_2_ adsorption is predominantly physisorption^64^. The obtained Arrhenius parameters can be used for predicting the adsorption rate constants at different operating temperatures.

For investigating the cyclic stability of MCC-K3, a repeated adsorption–desorption test was conducted by alternatingly switching the flowing gas between N_2_ and CO_2_ at a fixed temperature at 25 °C and ~1 bar, as shown in Fig. 6c. Adsorbed CO_2_ was easily desorbed simply by changing the gas flow from CO_2_ to N_2_, which was expected from the low heat of adsorption of MCC-K3. During 10 cycles, MCC-K3 was successfully regenerated by gas purging, with cyclic stability, retaining ~99% of the initial CO_2_ adsorption capacity.

## Conclusions

In this study, microporous carbon compartments (MCCs) were prepared from wheat flour via carbonization and KOH activation for the capture of CO_2_ by adsorption. The carbonization of pristine wheat flour induced the pyrolysis of non-carbon elements, developing big rooms, which can permit gas molecules to access micropores, and the textural properties of MCCs were further improved by chemical activation with KOH. The KOH/C ratio was varied from 1 to 5, and the effects of KOH/C ratio on the textural properties and CO_2_ adsorption performance were investigated. With increasing KOH/C ratio up to 3, narrow micropores of less than 0.8 nm were primarily developed, while larger micropores were also produced from pore enlargement with excess KOH. An optimal KOH/C ratio was clearly identified for activation for attaining the maximum CO_2_ adsorption capacity of MCC: MCC-K3 exhibited the highest CO_2_ adsorption capacity of 5.70 and 3.48 mol kg^−1^ at 0 and 25 °C, respectively. The CO_2_ adsorption capacity significantly depended on the volume of the narrow micropores with a pore size of less than 0.8 nm rather than the surface area or pore volume of larger pores. MCC-K3 also exhibited excellent cyclic stability with facile regeneration, high selectivity for CO_2_ over N_2_, and rapid adsorption rates. As compared to the pseudo-first-order model, the pseudo-second-order kinetic model described the experimental adsorption data methodically. The outstanding overall CO_2_ adsorption performance indicate that KOH-activated MCCs can be promising CO_2_ adsorbents.

## Methods

### Synthesis of MCC and KOH-activated MCCs

For preparing microporous carbon compartments (MCCs), first, pristine wheat flour was placed in a horizontal cylindrical furnace with an inner diameter of 50 mm and then heated at a rate of 5 °C min^−1^ up to 900 °C under N_2_. Heating was maintained for 2 h. The MCC thus obtained was ground with an agitator mortar.

For activation, MCC was mixed with the prepared KOH solution by continuous stirring at 60 °C for 2 h. The KOH to MCC weight ratio varied in the range from 1 to 5. The mixture was then dried at 110 °C overnight, and the dried material was activated at 700 °C for 1 h at a heating rate of 5 °C min^−1^ under N_2_. The resulting material was thoroughly washed with a 10% HCl solution for removing any inorganic salt and then washed with distilled water. KOH-activated MCC is denoted as MCC-K*x*, where *x* denotes the KOH/C ratio.

### Sample characterization

The surface morphologies of KOH-activated MCCs were analyzed by scanning electron microscope (SEM, S-4300, Hitachi) and high-resolution transmission electron microscope (HRTEM, G2 F30ST, Tecnai). The specific surface area was estimated by the Brunauer, Emmett, and Teller (BET) equation based on the information obtained by N_2_ adsorption at 77 K using a volumetric sorption analyzer (ASAP2020, Micromeritics). X-ray diffraction patterns were recorded with an X-ray diffractometer (XRD, X’ Pert MPD, Philips) using Cu Kα radiation in the 2θ range of 5–50°. A thermogravimetric analyzer (TGA, Q50, TA instruments) was used for measuring the weight loss of the pristine wheat flour at a heating rate of 10 °C min^−1^ under N_2_.

CO_2_ adsorption uptake was measured by the same volumetric sorption analyzer used for N_2_ adsorption isotherms. Prior to adsorption tests, samples were degassed at 150 °C under a vacuum (10 μm-Hg) for 12 h, and CO_2_ adsorption isotherms were obtained at four different temperatures of 0, 25, 50, and 75 °C. Cyclic adsorption stability was tested by TGA with CO_2_ adsorption at 25 °C and atmospheric pressure and desorption under N_2_ purge. CO_2_ adsorption was carried out for 40 min and the sample was regenerated using N_2_ purge for 90 min after each CO_2_ adsorption step. Also, CO_2_ adsorption kinetic studies were conducted by TGA, recording the weight change under CO_2_ for 3 h at temperatures of 30, 40, and 50 °C.

## Additional Information

**How to cite this article**: Hong, S.-M. *et al.* CO_2_ Capture in the Sustainable Wheat-Derived Activated Microporous Carbon Compartments. *Sci. Rep.* **6**, 34590; doi: 10.1038/srep34590 (2016).

## Supplementary Material

Supplementary Information

## Figures and Tables

**Table 1:** Textural properties and CO_2_ adsorption capacities at temperatures of 0, 25, 50, and 75 °C for MCC and KOH-activated MCCs.

Sample	Textural properties				CO_2_ adsorption capacity ^d)^ (mol kg^–1^)			
	S_BET_ (m^2^ g^−1^)	V_T_^a)^ (cm^3^ g^−1^)	V_m_^b)^ (cm^3^ g^−1^)	V_nm_^c)^ (cm^3^ g^−1^)	0 °C	25 °C	50 °C	75 °C
MCC	648	0.299	0.266	0.218	3.44	2.28	1.32	0.48
MCC-K1	916	0.432	0.367	0.250	3.82	2.42	1.41	0.78
MCC-K2	1057	0.581	0.474	0.271	4.41	2.77	1.60	0.78
MCC-K3	1438	0.654	0.581	0.389	5.70	3.48	1.98	1.08
MCC-K4	1801	0.840	0.704	0.355	5.29	3.13	1.73	0.84
MCC-K5	2192	1.076	0.786	0.282	4.42	2.56	1.38	0.76

^a)^Total pore volume at P/P_0_ ~0.99; ^b)^Micropore volume determined from the Dubinin–Radushkevich equation; ^c)^Cumulative narrow micropore volume calculated in the range of pore sizes up to 0.8 nm; ^d)^CO_2_ adsorption capacity measured under a pressure of ~1 bar.

**Table 2:** CO_2_ adsorption kinetic parameters for MCC-K3 using pseudo-first-order and pseudo-second-order models at temperatures of 30, 40, and 50 °C.

Temperature (°C)	Pseudo-first-order			Pseudo-second-order		
	*K*_1_ (min^−1^)	R^2^	*Err*%	*K*_2_ (mol kg^−1^ min^−1^)	R^2^	*Err*%
30	0.198	0.72	8.37	0.061	0.96	4.27
40	0.232	0.71	7.50	0.082	0.92	4.58
50	0.254	0.70	7.41	0.108	0.92	4.08

## References

[b1] D’Alessandro D. M., Smit B. & Long J. R. Carbon Dioxide Capture: Prospects for New Materials. Angew. Chem. Int. Ed. 49, 6058–6082 (2010). 10.1002/anie.201000431 20652916

[b2] Li B., Duan Y., Luebke D. & Morreale B. Advances in CO_2_ capture technology: A patent review. Appl. Energy 102, 1439–1447 (2013).

[b3] Cozier M. The UN COP21 Climate Change Conference and the role of CCS. Greenh. Gases 5, 697–700 (2015).

[b4] Yu K. M. K., Curcic I., Gabriel J. & Tsang S. C. E. Recent advances in CO_2_ capture and utilization. ChemSusChem 1, 893–899 (2008). 18985640 10.1002/cssc.200800169

[b5] MacDowell N. *et al.* An overview of CO_2_ capture technologies. Energy Environ. Sci. 3, 1645–1669 (2010).

[b6] Kenarsari S. D. *et al.* Review of recent advances in carbon dioxide separation and capture. RSC Adv. 3, 22739–22773 (2013).

[b7] Favre E. Carbon dioxide recovery from post-combustion processes: Can gas permeation membranes compete with absorption? J. Membrane Sci. 294, 50–59 (2007).

[b8] Samanta A., Zhao A., Shimizu G. K. H., Sarkar P. & Gupta R. Post-combustion CO_2_ capture using solid sorbents: A review. Ind. Eng. Chem. Res. 51, 1438–1463 (2012).

[b9] Choi S., Drese J. H. & Jones C. W. Adsorbent materials for carbon dioxide capture from large anthropogenic point sources. ChemSusChem 2, 796–854 (2009). 19731282 10.1002/cssc.200900036

[b10] Sayari A., Belmabkhout Y. & Serna-Guerrero R. Flue gas treatment via CO_2_ adsorption. Chem. Eng. J. 171, 760–774 (2011).

[b11] El Qada E. N., Allen S. J. & Walker G. M. Adsorption of Methylene Blue onto activated carbon produced from steam activated bituminous coal: A study of equilibrium adsorption isotherm. Chem. Eng. J. 124, 103–110 (2006).

[b12] Xia K., Gao Q., Jiang J. & Hu J. Hierarchical porous carbons with controlled micropores and mesopores for supercapacitor electrode materials. Carbon 46, 1718–1726 (2008).

[b13] Lu G. Q. & Do D. D. A kinetic study of coal reject-derived char activation with CO_2_, H_2_O, and air. Carbon 30, 21–29 (1992).

[b14] Kim M.-H., Kim K.-B., Park S.-M. & Roh K. C. Hierarchically structured activated carbon for ultracapacitors. Sci. Rep. 6, 21182 (2016). 26878820 10.1038/srep21182 PMC4754731

[b15] Lillo-Ródenas M. A., Cazorla-Amorós D. & Linares-Solano A. Understanding chemical reactions between carbons and NaOH and KOH: An insight into the chemical activation mechanism. Carbon 41, 267–275 (2003).

[b16] Hayashi J. i., Horikawa T., Takeda I., Muroyama K. & Nasir Ani F. Preparing activated carbon from various nutshells by chemical activation with K_2_CO_3_. Carbon 40, 2381–2386 (2002).

[b17] Azevedo D. C. S. *et al.* Microporous activated carbon prepared from coconut shells using chemical activation with zinc chloride. Microporous Mesoporous Mater. 100, 361–364 (2007).

[b18] Gupta V. K., Pathania D., Sharma S. & Singh P. Preparation of bio-based porous carbon by microwave assisted phosphoric acid activation and its use for adsorption of Cr(VI). J. Colloid Interface Sci. 401, 125–132 (2013). 23611657 10.1016/j.jcis.2013.03.020

[b19] Sevilla M. & Mokaya R. Energy storage applications of activated carbons: supercapacitors and hydrogen storage. Energy Environ. Sci. 7, 1250–1280 (2014).

[b20] Sun N. *et al.* Synthesis, characterization and evaluation of activated spherical carbon materials for CO_2_ capture. Fuel 113, 854–862 (2013).

[b21] Shen W., Zhang S., He Y., Li J. & Fan W. Hierarchical porous polyacrylonitrile-based activated carbon fibers for CO_2_ capture. J. Mater. Chem. 21, 14036–14040 (2011).

[b22] Li Y., Ben T., Zhang B., Fu Y. & Qiu S. Ultrahigh gas storage both at low and high pressures in KOH-activated carbonized porous aromatic frameworks. Sci. Rep. 3, 2420 (2013). 23939301 10.1038/srep02420 PMC3741621

[b23] Sevilla M., Parra J. B. & Fuertes A. B. Assessment of the role of micropore size and N-doping in CO_2_ capture by porous carbons. ACS Appl. Mater. Interfaces 5, 6360–6368 (2013). 23789916 10.1021/am401423b

[b24] Wahby A., Silvestre-Albero J., Sepúlveda-Escribano A. & Rodríguez-Reinoso F. CO_2_ adsorption on carbon molecular sieves. Microporous Mesoporous Mater. 164, 280–287 (2012).

[b25] Yao J. *et al.* Preparation of colloidal microporous carbon spheres from furfuryl alcohol. Carbon 43, 1709–1715 (2005).

[b26] Tao Y., Endo M., Inagaki M. & Kaneko K. Recent progress in the synthesis and applications of nanoporous carbon films. J. Mater. Chem. 21, 313–323 (2011).

[b27] Ngernyen Y., Tangsathitkulchai C. & Tangsathitkulchai M. Porous properties of activated carbon produced from eucalyptus and wattle wood by carbon dioxide activation. Korean J. Chem. Eng. 23, 1046–1054 (2006).

[b28] Lee J., Kim J. & Hyeon T. Recent progress in the synthesis of porous carbon materials. Adv. Mater. 18, 2073–2094 (2006).

[b29] Ferrero G. A., Fuertes A. B. & Sevilla M. From Soybean residue to advanced supercapacitors. Sci. Rep. 5, 16618 (2015). 26568473 10.1038/srep16618 PMC4645100

[b30] Unur E., Brutti S., Panero S. & Scrosati B. Nanoporous carbons from hydrothermally treated biomass as anode materials for lithium ion batteries. Microporous Mesoporous Mater. 174, 25–33 (2013).

[b31] Kante K., Nieto-Delgado C., Rangel-Mendez J. R. & Bandosz T. J. Spent coffee-based activated carbon: Specific surface features and their importance for H_2_S separation process. J. Hazard. Mater. 201–202, 141–147 (2012). 10.1016/j.jhazmat.2011.11.053 22154120

[b32] Sevilla M. & Fuertes A. B. Sustainable porous carbons with a superior performance for CO_2_ capture. Energy Environ. Sci. 4, 1765–1771 (2011).

[b33] Suhas, Carrott P. J. M. & Ribeiro Carrott M. M. L. Lignin – from natural adsorbent to activated carbon: A review. Bioresour. Technol. 98, 2301–2312 (2007). 17055259 10.1016/j.biortech.2006.08.008

[b34] Xu B., Hou S., Cao G., Wu F. & Yang Y. Sustainable nitrogen-doped porous carbon with high surface areas prepared from gelatin for supercapacitors. J. Mater. Chem. 22, 19088–19093 (2012).

[b35] Li M., Li W. & Liu S. Hydrothermal synthesis, characterization, and KOH activation of carbon spheres from glucose. Carbohydr. Res. 346, 999–1004 (2011). 21481847 10.1016/j.carres.2011.03.020

[b36] Kumar R. S. & Sakthivel N. Exopolysaccharides of Xanthomonas pathovar strains that infect rice and wheat crops. Appl. Microbiol. Biotechnol. 55, 782–786 (2001). 11525629 10.1007/s002530000563

[b37] Amir R. M. *et al.* Application of Fourier transform infrared (FTIR) spectroscopy for the identification of wheat varieties. J. Food Sci. Tech. Mys. 50, 1018–1023 (2013). 10.1007/s13197-011-0424-y PMC3722403 24426012

[b38] Flores-Morales A., Jiménez-Estrada M. & Mora-Escobedo R. Determination of the structural changes by FT-IR, Raman, and CP/MAS 13C NMR spectroscopy on retrograded starch of maize tortillas. Carbohydr. Polym. 87, 61–68 (2012). 10.1016/j.carbpol.2011.07.011 34663011

[b39] Robens E., Rouquerol F., Rouquerol J. & Sing K. Adsorption by Powders and Porous Solids, Vol. 13, 439–442 (Academic Press, 1999).

[b40] Wei H. R. *et al.* Granular bamboo-derived activated carbon for high CO_2_ adsorption: the dominant role of narrow micropores. ChemSusChem 5, 2354–2360 (2012). 23132775 10.1002/cssc.201200570

[b41] Li Y., Zou B., Hu C. & Cao M. Nitrogen-doped porous carbon nanofiber webs for efficient CO_2_ capture and conversion. Carbon 99, 79–89 (2016).

[b42] Alabadi A., Razzaque S., Yang Y., Chen S. & Tan B. Highly porous activated carbon materials from carbonized biomass with high CO_2_ capturing capacity. Chem. Eng. J. 281, 606–612 (2015).

[b43] Huang B., Shao H., Liu N., Xu Z. J. & Huang Y. From fish scales to highly porous N-doped carbon: a low cost material solution for CO_2_ capture. RSC Adv. 5, 88171–88175 (2015).

[b44] Wang J. *et al.* Fungi-based porous carbons for CO_2_ adsorption and separation. J. Mater. Chem. 22, 13911–13913 (2012).

[b45] Bae J.-S. & Su S. Macadamia nut shell-derived carbon composites for post combustion CO_2_ capture. Int. J. Greenh. Gas Control 19, 174–182 (2013).

[b46] Hao G.-P. *et al.* Structurally designed synthesis of mechanically stable poly(benzoxazine-co-resol)-based porous carbon monoliths and their application as high-performance CO_2_ capture sorbents. J. Am. Chem. Soc. 133, 11378–11388 (2011). 21692510 10.1021/ja203857g

[b47] Plaza M. G., González A. S., Pevida C., Pis J. J. & Rubiera F. Valorisation of spent coffee grounds as CO_2_ adsorbents for postcombustion capture applications. Appl. Energy 99, 272–279 (2012).

[b48] Nelson K. M. *et al.* Preparation and CO_2_ adsorption properties of soft-templated mesoporous carbons derived from chestnut tannin precursors. Microporous Mesoporous Mater. 222, 94–103 (2016).

[b49] Hu X., Radosz M., Cychosz K. A. & Thommes M. CO_2_-filling capacity and selectivity of carbon nanopores: synthesis, texture, and pore-size distribution from quenched-solid density functional theory (QSDFT). Environ. Sci. Technol. 45, 7068–7074 (2011). 21721529 10.1021/es200782s

[b50] Patino J. *et al.* DES assisted synthesis of hierarchical nitrogen-doped carbon molecular sieves for selective CO_2_*versus* N_2_ adsorption. J. Mater. Chem. A 2, 8719–8729 (2014).

[b51] Tseng R.-L., Wu F.-C. & Juang R.-S. Adsorption of CO_2_ at atmospheric pressure on activated carbons prepared from melamine-modified phenol–formaldehyde resins. Sep. Purif. Technol. 140, 53–60 (2015).

[b52] Ganesan A. & Shaijumon M. M. Activated graphene-derived porous carbon with exceptional gas adsorption properties. Microporous Mesoporous Mater. 220, 21–27 (2016).

[b53] Liu Y. Y. & Wilcox J. Molecular simulation studies of CO_2_ adsorption by carbon model compounds for carbon capture and sequestration applications. Environ. Sci. Technol. 47, 95–101 (2013). 22747244 10.1021/es3012029

[b54] Pan H., Ritter J. A. & Balbuena P. B. Examination of the approximations used in determining the isosteric heat of adsorption from the Clausius−Clapeyron equation. Langmuir 14, 6323–6327 (1998).

[b55] Ben T. *et al.* Selective adsorption of carbon dioxide by carbonized porous aromatic framework (PAF). Energy Environ. Sci. 5, 8370–8376 (2012).

[b56] Mahurin S. M., Górka J., Nelson K. M., Mayes R. T. & Dai S. Enhanced CO_2_/N_2_ selectivity in amidoxime-modified porous carbon. Carbon 67, 457–464 (2014).

[b57] Plaza M. G. *et al.* Developing almond shell-derived activated carbons as CO_2_ adsorbents. Sep. Purif. Technol. 71, 102–106 (2010).

[b58] Seema H. *et al.* Highly selective CO_2_ capture by S-doped microporous carbon materials. Carbon 66, 320–326 (2014).

[b59] To J. W. F. *et al.* Hierarchical N-doped carbon as CO_2_ adsorbent with high CO_2_ selectivity from rationally designed polypyrrole precursor. J. Am. Chem. Soc. 138, 1001–1009 (2016). 26717034 10.1021/jacs.5b11955

[b60] Montagnaro F. *et al.* Post-combustion CO_2_ adsorption on activated carbons with different textural properties. Microporous Mesoporous Mater. 209, 157–164 (2015).

[b61] Nan D., Liu J. & Ma W. Electrospun phenolic resin-based carbon ultrafine fibers with abundant ultra-small micropores for CO_2_ adsorption. Chem. Eng. J. 276, 44–50 (2015).

[b62] Sethia G. & Sayari A. Comprehensive study of ultra-microporous nitrogen-doped activated carbon for CO_2_ capture. Carbon 93, 68–80 (2015).

[b63] Hong S. M., Choi S. W., Kim S. H. & Lee K. B. Porous carbon based on polyvinylidene fluoride: Enhancement of CO_2_ adsorption by physical activation. Carbon 99, 354–360 (2016).

[b64] Creamer A. E., Gao B. & Zhang M. Carbon dioxide capture using biochar produced from sugarcane bagasse and hickory wood. Chem. Eng. J. 249, 174–179 (2014).

