# Supplementary material for: CO2 Capture in the Sustainable Wheat-Derived Activated Microporous Carbon Compartments
Source: Sci Rep. 2016 Oct 4;6:34590. doi: 10.1038/srep34590 (PMC5048159; doi:10.1038/srep34590)
Supplement: Supplementary Information [file srep34590-s1.pdf]

Supplementary information for *Scientific Reports*

CO_2 Capture in the Sustainable Wheat-Derived Activated Microporous Carbon Compartments

Seok-Min Hong[1], Eunji Jang[1], Arthur D. Dysart[2], Vilas G. Pol[2,*], Ki Bong Lee[1,*]

[1] Department of Chemical and Biological Engineering, Korea University, 145 Anam-ro, Seongbuk-gu, Seoul 136-713, Republic of Korea

[2] School of Chemical Engineering, Purdue University, 480 Stadium Mall Drive, West Lafayette, Indiana 47907-2100, United States

[1,*] Tel.: +82 2 3290 4851; FAX: +82 2 926 6102; E-mail: kibonglee@korea.ac.kr

[2,*] Tel: +01 765 494 0044; FAX: +01 765 494 0805; E-mail: vpol@purdue.edu

Clausius–Clapeyron equation

The isosteric heat of adsorption was calculated by CO_2 adsorption isotherms measured at different temperatures.

$$\Delta H = -R\left[\frac{\partial \ln(p)}{\partial(1/T)}\right]_n$$

Here, *R* represents the universal gas constant; *T*, the absolute temperature; *p*, the pressure; and *n*, the amount of adsorbed CO_2.

Ideal adsorption solution theory

Adsorption selectivity ($S_{i,j}$) for a binary mixture of CO_2 and N_2 was calculated by the ideal adsorption solution theory (IAST) as follows:

$$S_{CO_2,N_2} = \frac{q_{CO_2} / q_{N_2}}{p_{CO_2} / p_{N_2}}$$

Here, q_{CO_2} and q_{N_2} represent adsorption uptake of CO_2 and N_2; p_{CO_2} and p_{N_2} represent the partial pressures of CO_2 and N_2, respectively.

Model validity evaluation

For evaluating the accuracy of pseudo-first-order and pseudo-second-order models, the normalized standard deviation ($\Delta Err\%$) was calculated as follows:

$$\Delta Err\% = \sqrt{\frac{\sum[(q_{t,mes} - q_{t,est}) / q_{t,mes}]^2}{n - 1}} \times 100$$

Here, *n* represents the total number of experimental adsorption points. $q_{t,mes}$ and $q_{t,est}$ represent the measured and estimated adsorption capacities, respectively.

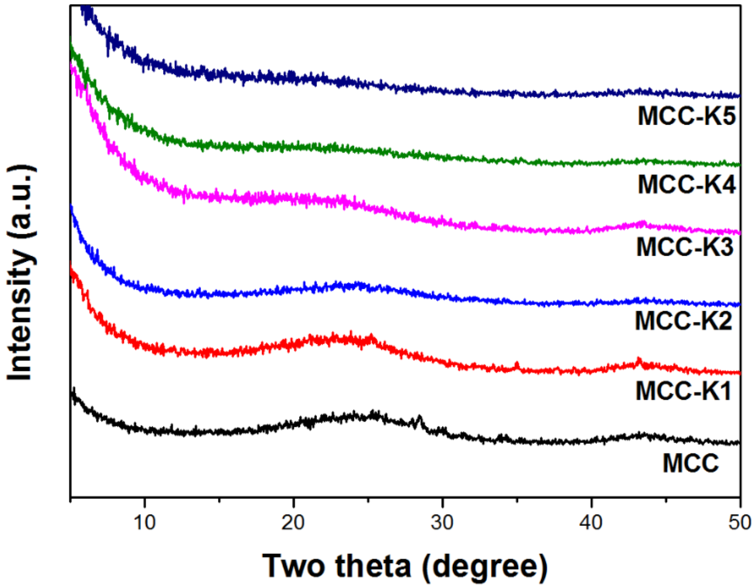

Figure S1. XRD patterns of MCC and KOH-activated MCCs.

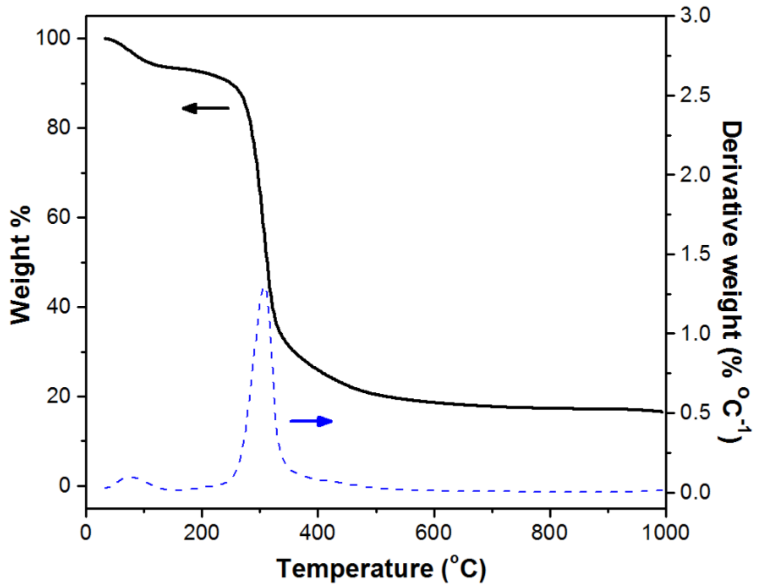

Figure S2. Weight change of pristine wheat flour with increasing temperature under N_2.

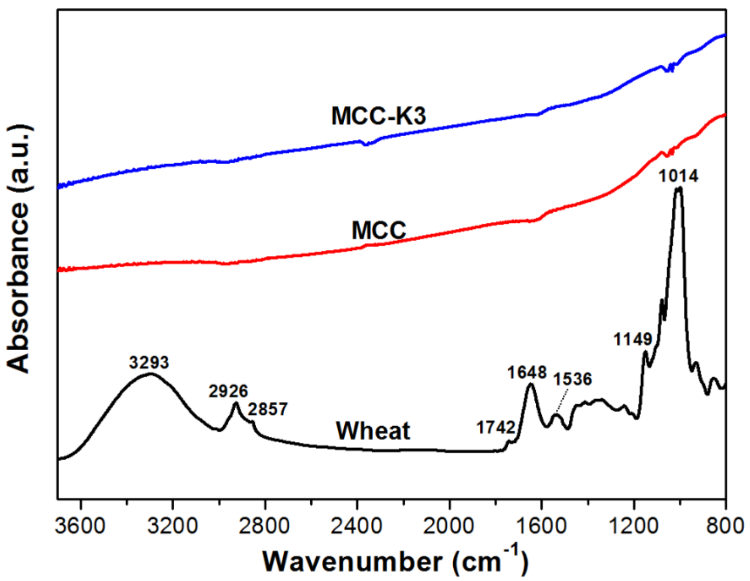

Figure S3. FTIR spectra of pristine wheat flour, MCC, and MCC KOH-activated with a KOH/C ratio of 3.

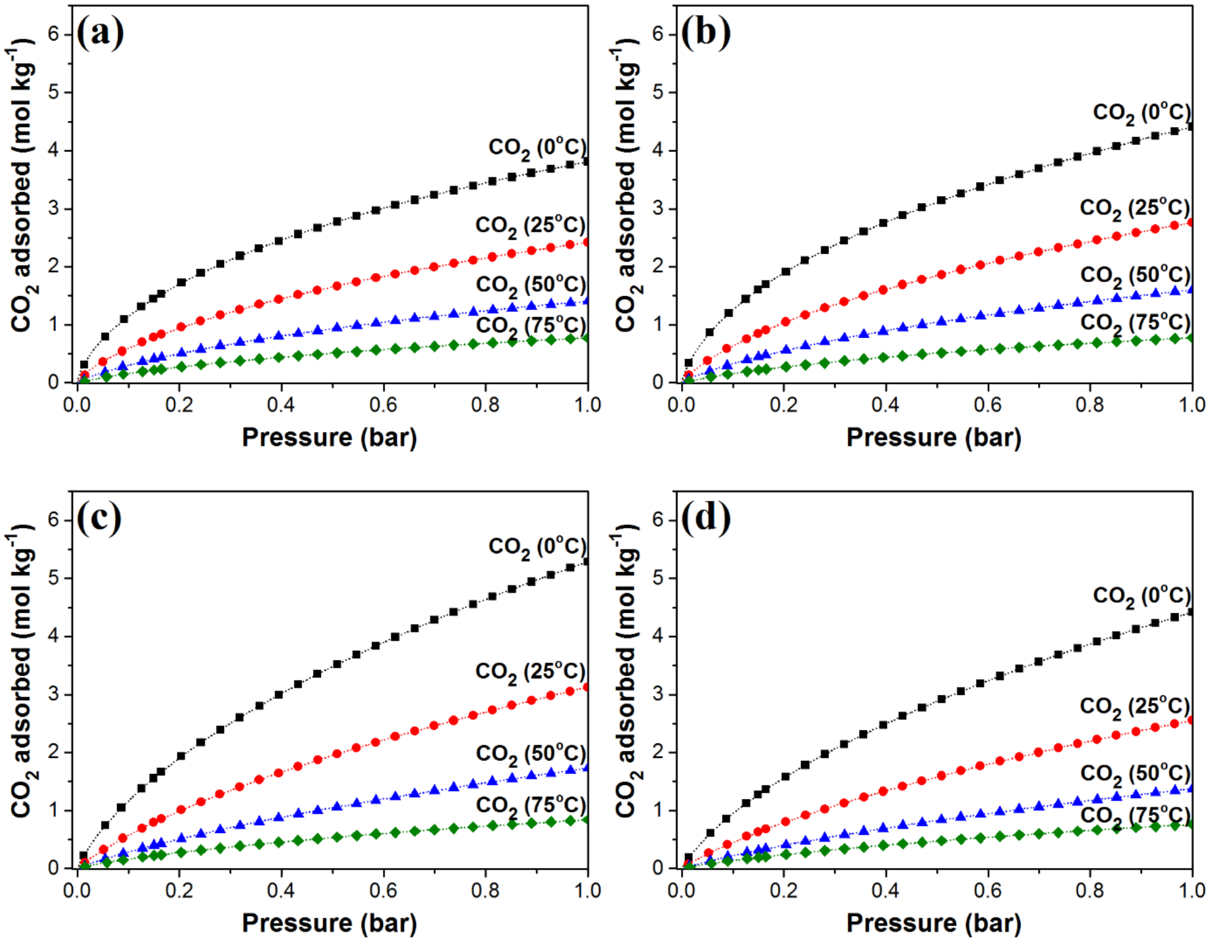

Figure S4. CO_2 adsorption isotherms of KOH-activated MCCs with KOH/C ratios of (a) 1, (b) 2, (c) 4, and (d) 5. Symbols and dashed lines represent experimental data and fitted results, respectively.

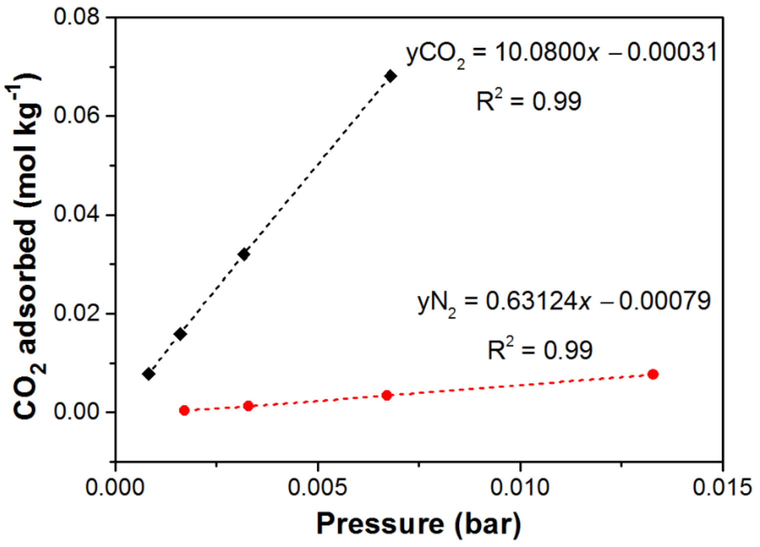

Figure S5. Initial slopes from the CO_2 and N_2 isotherms at 25 °C for MCC KOH-activated with a KOH/C ratio of 3.

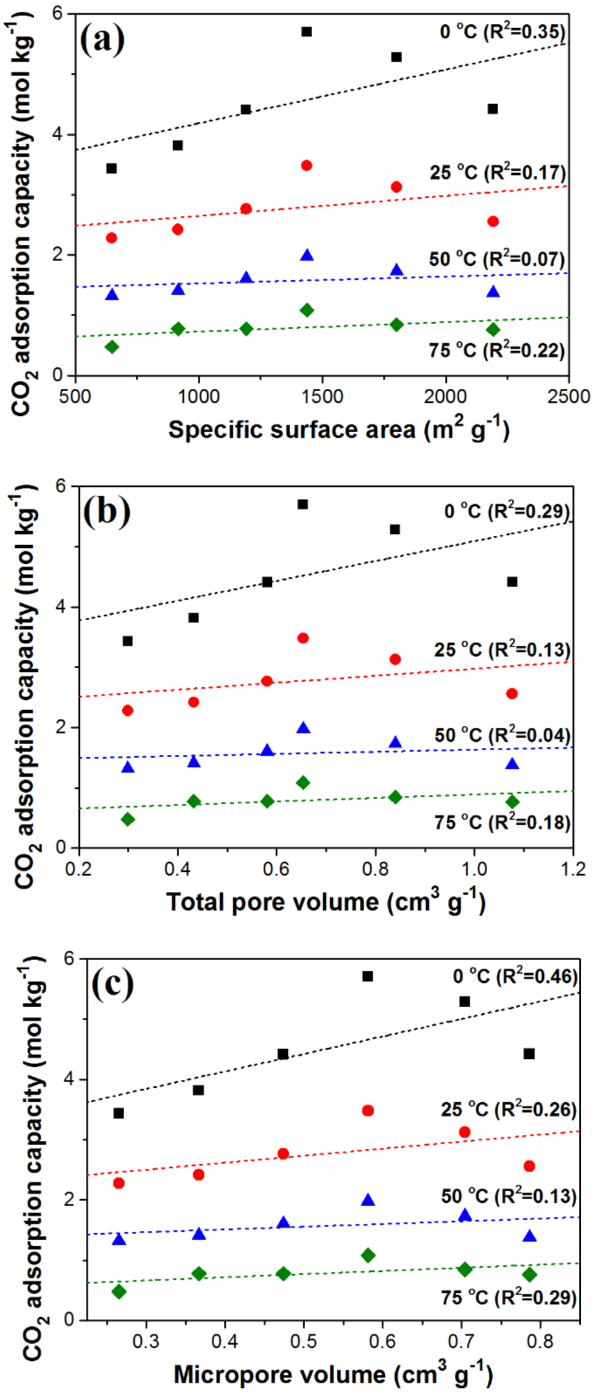

Figure S6. Correlation between CO_2 adsorption capacities and textural properties; (a) specific surface area, (b) total pore volume, and (c) micropore volume.

Table S1. Comparison of CO_2 adsorption capacities measured at a pressure of ~1 bar and CO_2/N_2 selectivities for different carbonaceous adsorbents.

Sample	CO_2 adsorption capacity at 25 °C (mol kg^{-1})	Selectivity of CO_2 over N_2 at 25 °C	Reference
**Microporous carbon compartment derived from wheat**	**3.48**	**15 [(a)] / 16 [(b)]**	**This work**
N-doped porous carbon nanofiber	4.42	26 [(a)]	[41]
N-enriched porous carbon derived from biomass	3.84	51 [(b)]	[42]
N-doped porous carbon based on fish scale	3.73	4.6 [(c)]	[43]
Fungi-based porous carbon	3.5	18.5 [(b)]	[44]
Carbon composites derived from macadamia nut shell	3.48	13 [(b)]	[45]
CO_2-activated poly(benzoxazine-co-resol)-based porous carbon monolith	3.3	28 [(b)]	[46]
Microporous carbon derived from coffee grounds	3.0	5 [(c)]	[47]
NH_3-activated chestnut tannin-based mesoporous carbon	2.86	28.1 [(b)]	[48]
Porous carbon derived from petroleum coke	2.73	10 [(c)]	[49]
Hierarchical N-doped carbon molecular sieve	2.7	5.4 [(a)]	[50]
Steam-activated carbon derived from melamine-modified phenol-formaldehyde resins	2.2	-	[51]
Activated graphene-derived porous carbon	2	-	[52]

[a)] Based on the ideal adsorption solution theory (IAST) with CO_2/N_2 = 15:85. [b)] Based on the initial slope of adsorption isotherm. [c)] Based on the CO_2 and N_2 adsorption capacities at ~1 bar.
